# Three-dimensional analysis of nuclear heterochromatin distribution during early development in the rabbit

**DOI:** 10.1007/s00412-018-0671-z

**Published:** 2018-04-18

**Authors:** Amélie Bonnet-Garnier, Kiên Kiêu, Tiphaine Aguirre-Lavin, Krisztina Tar, Pierre Flores, Zichuan Liu, Nathalie Peynot, Martine Chebrout, András Dinnyés, Véronique Duranthon, Nathalie Beaujean

**Affiliations:** 1 UMR BDR, INRA, ENVA, Université Paris Saclay, 78350 Jouy-en-Josas, France; 2 UR341 MaIAGE, INRA, Université Paris Saclay, 78350 Jouy-en-Josas, France; 3 Present Address: Department of Medical Chemistry, Faculty of Medicine, University of Debrecen, Debrecen, Hungary; 4 BioTalentum Ltd., Aulich Lajos str. 26, Gödöllő, 2100 Hungary; 5 Present Address: Friedrich Miescher Institute for Biomedical Research, Basel, Switzerland; 6 State Key Laboratory of Reproductive Biology, Institute of Zoology, Chinese Academy of Sciences, Beijing, 100101 China; 7 Present Address: Univ Lyon, Université Claude Bernard Lyon 1, Inserm, INRA, Stem Cell and Brain Research Institute U1208, USC1361, 69500 Bron, France

**Keywords:** Embryos, 3D-FISH, Centromeres, Epigenetic modifications, Satellite sequences

## Abstract

Changes to the spatial organization of specific chromatin domains such as constitutive heterochromatin have been studied extensively in somatic cells. During early embryonic development, drastic epigenetic reprogramming of both the maternal and paternal genomes, followed by chromatin remodeling at the time of embryonic genome activation (EGA), have been observed in the mouse. Very few studies have been performed in other mammalian species (human, bovine, or rabbit) and the data are far from complete. During this work, we studied the three-dimensional organization of pericentromeric regions during the preimplantation period in the rabbit using specific techniques (3D-FISH) and tools (semi-automated image analysis). We observed that the pericentromeric regions (identified with specific probes for Rsat I and Rsat II genomic sequences) changed their shapes (from pearl necklaces to clusters), their nuclear localizations (from central to peripheral), as from the 4-cell stage. This reorganization goes along with histone modification changes and reduced amount of interactions with nucleolar precursor body surface. Altogether, our results suggest that the 4-cell stage may be a crucial window for events necessary before major EGA, which occurs during the 8-cell stage in the rabbit.

**Electronic supplementary material:**

The online version of this article (10.1007/s00412-018-0671-z) contains supplementary material, which is available to authorized users.

## Introduction

The nucleus of a cell is divided into several membrane-less compartments such as the nucleolus or nuclear speckle (Dundr 2012) that are dedicated to specific nuclear functions (rRNA expression and processing or RNA splicing) (Dundr and Misteli 2010; Sawyer et al. 2016). During the past 10 years, the involvement of nuclear architecture in the regulation of gene expression, and more particularly the spatial organization of chromatin, have been studied intensively (for a review, see Schneider and Grosschedl 2007; Joffe et al. 2010; Bickmore and van Steensel 2013; Pombo and Dillon 2015; Dekker and Heard 2015).

Especially, the genome is organized in chromosomes that occupy specific chromosome territories (CTs) in the interphase nucleus. In somatic cells, these CTs are not arranged at random (Cremer and Cremer 2010), and it is generally accepted that gene-poor chromosomes are located at the periphery of the nucleus while their gene-rich counterparts are found in the center (Croft et al. 1999; Boyle et al. 2001; Hübner and Spector 2010). In addition, some specific genomic regions (such as gene clusters) tend to change their three-dimensional (3D) position in correlation with their transcription status (Lanctot et al. 2007; Therizols et al. 2014) and may even loop out of the CTs (Volpi et al. 2000; Chambeyron and Bickmore 2004).

The interphase chromatin comprises open regions that permit transcription (the euchromatin) or dense and compact regions that allow little or no transcription (the heterochromatin) (Zinner et al. 2006; Joffe et al. 2010; Politz et al. 2013). In somatic or embryonic stem cells, heterochromatin is principally located at the nuclear envelope or around the nucleolus and generally corresponds to telomeric, centromeric, and pericentromeric regions. In numerous species (human, mouse, chicken, bovine), the centromeric and pericentromeric heterochromatin of several chromosomes cluster to form specific structures called chromocenters (Haaf and Schmid 1991; Guenatri et al. 2004; Maslova et al. 2015). These highly condensed pericentromeric regions, intensely labeled with 4′,6-diamidino-2-phenylindole (DAPI) dye, are mostly composed of A-T-rich DNA sequences (called major and minor satellites in the mouse; Vissel and Choo 1989; Lehnertz et al. 2003) and are associated with repressive histone marks: such as histone H4 trimethyl Lys20 (H4K20me3) and histone H3 trimethyl Lys9 (H3K9me3) which attracts the heterochromatin-specific protein HP1 through its chromodomain (Peters et al. 2001, 2003; Maison and Almouzni 2004). Similarly, centromeric regions are characterized by specific centromeric proteins (CENPs) (Cheutin et al. 2003).

Changes to the spatial organization of these chromatin compartments (chromosome, gene clusters, or heterochromatin) have been observed during differentiation (Solovei et al. 2009) or senescence (Chandra and Narita 2013), but most work to date has been performed in somatic cells. Indeed, only a few studies have been carried out in the context of early development on bovine or rabbit embryos (Koehler et al. 2009; Pichugin et al. 2010; Yang et al. 2013; Popken et al. 2014a, b; review in Borsos and Torres-Padilla 2016).

Early embryonic development in mammals, from fertilization to implantation, is a particularly critical period. The newly formed zygote needs to remodel the highly specialized parental chromatin and switch on its own transcription within a very short time frame (Schultz et al. 1999), an event referred to as embryonic genome activation (EGA) which occurs at different points depending on the species (review in Schultz and Heyner 1992; Telford et al. 1990): at the 2-cell stage in the mouse (Moore 1975; review in Schultz 1993), at the 4- to 8-cell stages in humans (Braude et al. 1988), at the 8-cell stage in the bovine (Camous et al. 1984, 1986; Memili and First 1998; Graf et al. 2014), and between 8- and 16-cell stages in the rabbit (Manes 1973; Brunet-Simon et al. 2001).

This preimplantation period (from zygote to blastocyst) is characterized by major changes: epigenetic reprogramming, the onset of transcription, transition from a totipotent to a pluriporent state, and initial differentiations. A body of evidence has shown that these processes are accompanied by a drastic reorganization of nuclear architecture. Indeed, several studies on early embryonic development in the mouse (Martin et al. 2006a, b; Probst et al. 2007; Aguirre-Lavin et al. 2012) have demonstrated that the pericentromeric heterochromatin organization is dramatically modified during EGA and that these changes are essential for the embryo to fully develop to term (Maalouf et al. 2009; Probst et al. 2010). Moreover, specific structures called NPBs (nucleolar precursor bodies) appear to play an important role (review by Fulka and Aoki 2016). Firstly, at the 1-cell stage, several teams have suggested that NPBs ensure the correct remodeling of heterochromatin regions composed of “major satellite” sequences (Ogushi and Saitou 2010; Jachowicz et al. 2013; Fulka and Langerova 2014; Kyogoku et al. 2014). Secondly, these NPBs serve as a platform for the development of functional nucleoli from the end of the 2-cell stage through the morula stage (Zatsepina et al. 2003; Lavrentyeva et al. 2015; Koné et al. 2016). In the mouse, the removal of nucleolus-like structures from the growing oocyte, or the removal of NPBs at the start of the 1-cell stage, can lead to an arrest of development at the 2-cell stage. In addition, the organization of centromeric and pericentromeric sequences is disturbed and they display architectural defects (Fulka and Langerova 2014; Kyogoku et al. 2014).

In rabbit, Baran et al. (1997) identified by electron microscopy NPBs as round shape and compact structures from the 1- to 8-cell stages. At the 16-cell stage, some NPBs (called A-NPB) displayed a less compact structure associated with RNA detection but fully functional nucleoli were only detected at the morula and blastocyst stages (Baran et al. 1997). However, very few information are available concerning the nuclear organization in 3D (Yang et al. 2013; Popken et al. 2016) and the epigenetic marks associated with pericentromeric heterrochromatin (Brero et al. 2009; Reis e Silva et al. 2011; Salvaing et al. 2016) during early embryonic development in rabbit. In our previous study of heterochromatin organization (Yang et al. 2013), the HP1β and CENP patterns observed using immunodetection were seen to change at the time of EGA (8-cell stage).

However, chromocenters were not easily detected with these two proteins, so we decided to complete our study using probes specific to pericentromeric sequences during early embryonic development in the rabbit. We then examined the organization of constitutive heterochromatin in relation to EGA. Unlike the mouse, the remodeling of maternal and paternal genomes that occurs during the two first cell cycles after fertilization (Fulka et al. 2008) can be distinguished from the EGA at the 8-cell stage, making the rabbit an interesting model. We took advantage of the identification of two families of DNA repeat sequences (named Rsat I and Rsat II) which both localize to pericentromeric regions (Ékes et al. 2004). The Rsat I sequence comprises 375-bp-long repeat units while Rsat II is composed of repeat units between 585 and 590 bp (Ékes et al. 2004). Rsat I and Rsat II hybridized to the pericentromeric/centromeric regions of 11 and 12 different chromosomes pairs, respectively, and had nine chromosome pairs in common. To investigate the spatial distribution of pericentromeric/centromeric heterochromatin, we studied the three-dimensional distribution of Rsat I/Rsat II sequences in the nucleus using the fluorescent in situ hybridization (3D-FISH) technique over the course of preimplantation development on whole-mount rabbit embryos (protocol adapted from that described by Aguirre-Lavin et al. 2012). We therefore used a semi-automated approach developed previously by our team (Ballester et al. 2008; Andrey et al. 2010) to investigate the morphometric features of pericentromeric/centromeric heterochromatin and determine their spatial distribution in the nucleus from confocal image stacks. We also studied their localization relative to the NPBs and determined the distribution of two epigenetic marks (H3K9me3 and H4K20me3) which classically characterize pericentromeric heterochromatin.

## Material and methods

### Ethics

Animal care and handling were carried out according to European regulations on animal welfare. ABG, NB, and VD are authorized to work with laboratory animals by Departmental Veterinary Regulatory Services (Nos. 78–184, 78–95, and, 78–101, respectively). This work was approved by the local Ethics Committee (Comethea Jouy-en-Josas/AgroParisTech accreditations 12/107 and 15–59 for in vitro analyses).

### Recovery of rabbit embryos and culture

Embryos were obtained from mature female New Zealand white rabbits after in vivo fertilization. Superovulation in the female rabbits was induced by five subcutaneous injections of pFSH (Stimufol®, Merial, France) during the 3 days prior to mating (two 5-μg doses on the first day at 12-h intervals, two 10-μg doses on the second day at 12-h intervals, and one 5-μg dose on the third day), followed 12 h later by an intravenous administration of 30 IU HCG (Chorulon, MSD Animal Health, USA) at the time of natural mating with male New Zealand white rabbits (Reis e Silva et al. 2011; Salvaing et al. 2016). The zygotes were flushed from the oviducts with PBS at 19 h post-coitum (hpc) and cultured in vitro up to different stages in M199 (Sigma-Aldrich, USA) supplemented with 2.5% fetal calf serum under mineral oil (Sigma-Aldrich, USA) in an incubator at 38.5 °C under 5% CO_2_ in air. The embryos were fixed and processed at 19 or 22 hpc (1-cell stage), 27 hpc (2-cell stage), 34 hpc (4-cell stage), 42 hpc (early 8-cell stage), 45 hpc (mid 8-cell stage), 48 hpc (late 8-cell stage), and 58 hpc (16-cell stage). All the embryos were then incubated in 5 mg/ml Pronase (Sigma-Aldrich, USA) at 37 °C for 5 to 10 min and transferred to HEPES medium (Sigma-Aldrich, USA) at 37 °C; then, mechanic removal of the zona pellucida was achieved by successive passages through a very fine pipette.

### Antibodies

HP1β was detected with a mouse monoclonal antibody (clone 1 MOD 1A9, Euromedex, 1:250 diluted in 2% bovine serum albumin (BSA) in PBS), using a rhodamine (TRITC)-conjugated anti-mouse secondary antibody (#715–025-151, Jackson ImmunoResearch, USA). The centromeres were labeled with a human CREST antibody which mostly recognizes CENP-A (Immunovision, Cellon Sarl, 1:250 in 2% BSA/PBS), using FITC-conjugated anti-human secondary antibody (#709–095-149, Jackson ImmunoResearch, USA). H3K9me3 and H4K20me3 were detected with rabbit polyclonal antibodies (39161 from Active Motif and ab9053 from Abcam, diluted 1:500 in 2% BAS/PBS) using a Cy5-conjugated anti-rabbit secondary antibody (#711–175-152, Jackson ImmunoResearch, USA). All the Jackson ImmunoResearch secondary antibodies raised in donkey were used at a dilution of 1:200 in 2% BSA/PBS.

### Immunofluorescence staining

The embryos thus harvested were processed for HP1β and CENPs, H3K9me3, or H4K20me3 immunostaining, as previously described (Pichugin et al. 2010; Yang et al. 2013). Briefly, the embryos were fixed in 2% paraformaldehyde (PAF; Sigma-Aldrich, USA) in PBS at RT for 20 min and permeabilized with 0.5% Triton X-100 (Sigma-Aldrich, USA) in PBS for 30 min on a heating plate at 25 °C. They were then blocked for 1 h at RT with 2% BSA/PBS and incubated with the primary antibodies overnight at 4 °C. After three washes with 0.05% Tween-20 (Sigma-Aldrich, USA) in PBS (15 min each), the embryos were incubated with the secondary antibodies (1 h, RT) then washed again and post-fixed for 10 min using 2% PFA. If any histone modifications were detected, DNA was counterstained with DAPI (Invitrogen) at 10 μg/ml. For microscopic observations, the embryos were mounted onto glass slides in Vectashield anti-fading agent (Vector Laboratories, USA).

### 3D-FISH experiments

FISH experiments were performed using DNA probes specific to the Rsat I and Rsat II sequences (Ékes et al. 2004) according to our standard protocol (Maalouf et al. 2009; Aguirre-Lavin et al. 2012) on 3D-preserved embryos.

The Rsat I and Rsat II probes were amplified by PCR on genomic rabbit DNA with the following primers: 5′ GAACAGGAAGATTGTGGTT 3′ and 5′ ATGTGTGGAGGATTTGA CTC 3′ (Rsat I) and 5′ ACTCAGACCCAGAAAACATTA 3′ and 5′ CTTAGAAATCTA CAGGTAACACGAC 3′ (Rsat II) according to the method described by Ékes et al. (2004). They were then labeled with Cy3 (Rsat I) or Cy5 (Rsat II) by random labeling (Invitrogen Kit, ref. 18095–011).

Unless otherwise specified, all steps were performed at room temperature (RT). After removal of the zona pellucida, the embryos were rinsed in HEPES medium (Sigma-Aldrich, USA), fixed in 4% PFA for 30 min, rinsed in PBS, and gently deposited with a minimum amount of PBS on a microscope slide to enable adherence (Menzel Superfrost Plus, Thermo Scientific). They were then fixed again in 4% PFA for 30 min, permeabilized for 30 min in 0.5% Triton X-100, and rinsed once for 5 min in 2× saline-sodium citrate (2×SSC) pH 6.3. RNA digestion was performed by incubation in 200 μg/ml RNase (Sigma, USA) in 2×SSC for 30 min at 37 °C. After two rinses in 2×SSC (5 min each) at room temperature, the slide was equilibrated in a hybridization buffer (50% formamide, SCC 2×, Denhardt 1×, 40 mM NaH_2_PO_4_, 10% dextran sulfate) for 1 to 2 h. The probe mix (1 μl Cy3-Rsat I solution (145 ng/μl), 2 μl Cy5 Rsat II solution (87 ng/μl) completed to 20 μl with hybridization buffer) and the slide were denatured separately for 10 min at 85 °C. A drop of hybridization mix containing the probes was then deposited on the slide, which was placed for 24 h at 37 °C in a humidified chamber. The samples were rinsed twice with 2×SSC at 42 °C and post-fixed in 2% PFA for 15 min. DNA was counterstained with YoproI (Molecular Probes, 1 μM), and Vectashield (Vector Laboratories, USA) was used as the mounting medium.

### Fluorescence microscopy

The embryos were examined with a ZEISS LSM 510 or 700 confocal laser scanning microscope (MIMA2 Platform, INRA). Observations were made using a 63× oil-immersion objective (NA 1.4). *Z* stacks were acquired with a frame size of 512 × 512 or 1024 × 1024, a pixel depth of 8 bits, and a *z* distance of 0.37 μm between optical sections. Fluorescence wavelengths of 405, 488, 555, and 639 nm were used to excite DAPI, YoProI or Alexa-488, Cy3, and Cy5, respectively.

### Image and statistical analyses

All embryos were analyzed visually with LSM510 or Zen software (Zeiss), step-by-step through the confocal *z* stacks and with the help of 3D reconstructions using AMIRA software. Except for the 1-cell stage embryos, which displayed a peculiar nuclear organization, we analyzed all the preimplantation embryos using the semi-automated image processing and analytical tools described below.

Three-dimensional images of nuclei acquired with the LSM510 software and saved as lsm files were processed using the ITK library (Yoo et al. 2002) and its Python interface (Lehmann et al. 2006).

Nuclear volumes were segmented for both CENP and Rsat images. Rsat spots were segmented in Rsat images. The HP1ß signal was smoothed before thresholding using several standard filters (median, Gaussian, opening/closing, gray hole filling). Thresholds for CENP images were determined using the RATS method (Kittler et al. 1985). As for Rsat images, thresholds were computed using the maximum entropy or Otsu method. Post-processing was performed in order to remove any masks that were too small or over-truncated (by the image boundary). Merged masks in CENP images were separated by applying a watershed transform on distance maps.

In order to quantify the radial position of non-segmented signals, a variant of the eroded volume fraction (EVF) was derived from the work by Ballester et al. (2008). In the original method, the EVF of a point within a nucleus is defined as the fraction of nuclear volume lying between that point and the nuclear membrane. The EVF rises from 0 for a signal at the nuclear periphery to 1 for a signal at the nuclear center. The EVF of points uniformly distributed within a nucleus is uniformly distributed between 0 and 1, and this property holds for any shape of the nucleus. In our study, we divided the nucleus into fractions with identical volumes, such that the mean EVF in each fraction increased linearly as the fractions were closer to the nuclear center and farther from the nuclear periphery. Then, for each fraction, we determined the proportion of the respective Rsat signals relative to the total signal in the nucleus, and compared the cumulative distribution obtained with the ideal case where the signal was uniformly distributed within the nucleus. In that case, the EVF distribution was exactly the identity function on the [0,1] interval, with 0 referring to the nuclear periphery. For each nucleus, the deviation from the uniform distribution was measured by the largest signed difference *d*_max_ between the two distributions for all the fractions. *d*_max_ values were between − 1 and 1, with positive values indicating a bias towards a more peripheral signal, and negative values a more central one. The higher the absolute *d*_max_ value is, the stronger the distribution bias is. The EVF method was obtained from the ITK library using 1000 fractions, and the results were analyzed using R.

Rsat spot segmentation started with a de-noising stage based on a Gaussian filter and a white tophat filter on volumes (high-pass filtering). Segmentation was performed using a standard white tophat filter, the height being defined as a quarter of the maximum intensity of the Rsat signal within the nuclear mask. Post-processing was applied to remove segmented spots below a size threshold so that no account would be taken of any false positives.

Assessing the polarity of Rsat spots (after segmentation) was based on the distance between the nuclear centroid and the centroid of all Rsat spots. Because this measurement depended on the size and shape of the nucleus, we applied the shape normalization method implemented in the ITK library. For each nucleus, we compared the distance between centroids measured to a uniform distribution of distances, these being were generated by simulation (500 random patterns of spots simulated per nucleus). A one-sample *t* test was performed between the distance measured and the simulated data, and the resulting *p* value was computed. The distribution of the *p* values obtained for the Rsat I signal were compared at each cell stage. A uniform distribution of *p* values (between 0 and 1) should indicate a random distribution of the spots in the nucleus. Small *p* values indicate great distances between nucleus and spot centroids and allow us to conclude to a polarity of the spots. *P* values close to 1 indicate small distances between centroids corresponding to a central position of the spots.

All statistical analyses and tests were performed using the associated R packages (R Development Core Team 2008). The normality and homogeneity of variances were tested using the “shapiro.test” and “bartlett.test” R packages, respectively, and linear models (“lm” or “glm” packages) with one fixed factor (stage) and one covariate were used to perform variance analysis throughout the period of development.

## Results

### Distribution and organization of Rsat I/Rsat II sequences in somatic cells

**Fig. 1:**
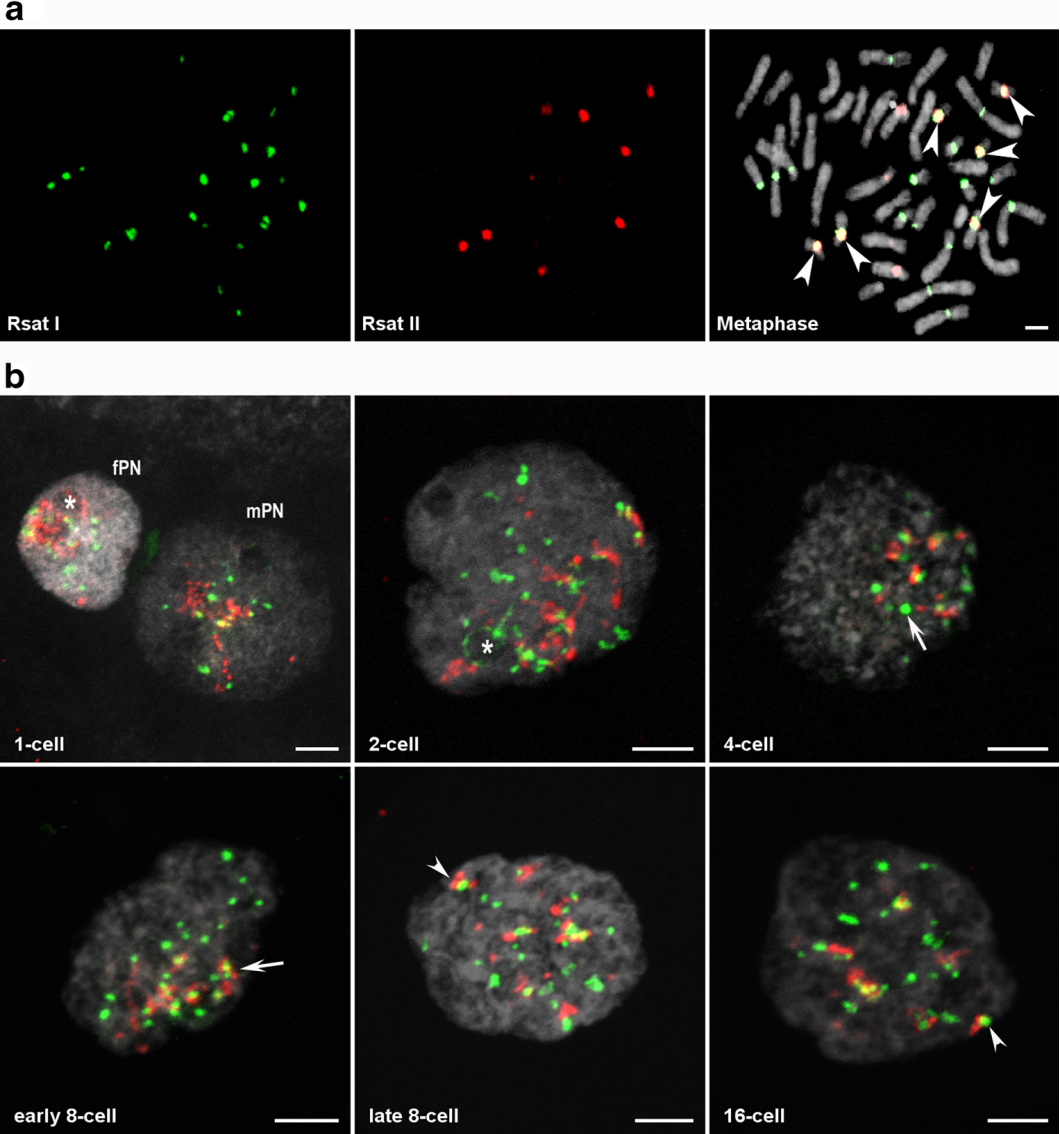
Localization of Rsat I/Rsat II DNA FISH signals on metaphase (**a**) and at different stages of rabbit preimplatation development (**b**). 2D or 3D-FISH were performed using specific probes against Rsat I (green) and Rsat II (red) sequences. DNA was counterstained with DAPI or Yopro-1 (gray). **a** Distribution of Rsat I/Rsat II FISH signals on the chromosome spread. The FISH signals of 24 Rsat I spots and 12 Rsat II spots were observed in the centromeric and pericentromeric regions of 12 chromosome pairs. Arrowheads indicate chromosomes labeled with both Rsat signals. Scale bar = 1 μm. **b** *Z* maximal projection of representative images of a nucleus from embryos fixed at the 1-cell stage (19 h post-coitum (hpc)) with female and male pronuclei (fPN and mPN), and the 2-cell (24 hpc), 4-cell (34 hpc), early and late 8-cell (42 and 49 hpc, respectively), and 16-cell (58 hpc) stages. Asterisks indicate NPBs associated with Rsat I/Rsat II FISH signals. Arrow and arrowheads indicate foci of Rsat FISH signals that are aggregated. Scale bar = 5 μm. Images were adjusted for brightness/contrast settings in each individual channels using ImageJ

We first tested Rsat I/Rsat II labeled with two different dyes (Cy3 and Cy5, respectively) and performed dual-color FISH on rabbit fibroblasts, either on metaphase spreads or on interphase 3D-preserved nuclei (Fig. 1a and Fig. S1a). We observed that in metaphase (2*n* = 44) of rabbit fibroblasts (Fig. 1a**)**, 11 chromosome pairs were labeled with Rsat I, one of these pairs having spots on both side of the centromere and the other chromosome pairs only displaying spots on one side. Six chromosome pairs were labeled with Rsat II: four pairs exhibited very strong signals and two pairs had weaker signals. In total, we detected 24 Rsat I and 12 Rsat II spots (Fig. 1a). Seven autosome pairs and the sexual chromosomes were not labeled with the probes used, similar to the findings of Ékes et al. (2004), and three chromosome pairs were labeled with both Rsat I and Rsat II sequences (arrowhead in Fig. 1a). During the interphase nucleus of a rabbit fibroblast, the sequences were clustered and mostly observed **(**Fig. S1a) at the nuclear periphery near the envelope.

### Nuclear organization and dynamics of Rsat I/Rsat II sequences in 1-cell through 16-cell stage embryos

To determine whether Rsat I and Rsat II sequences are reorganized during EGA, we performed 3D-FISH experiments on whole-mount rabbit embryos from fertilization through the 16-cell stage (Fig. 1b). In all FISH experiments on rabbit embryos, particular attention was paid to preserving the three dimensions of the nucleus, as illustrated in Fig. S1b. At the 1-cell (zygotic) stage we observed that Rsat I and Rsat II sequences are highly decondensed and formed pearl necklace-like structures that partially surround nucleolar precursor bodies (NPBs), in both male and female pronuclei (mPN and fPN). Moreover, Rsat II sequences appear even more decondensed than Rsat I sequences at this stage (arrow in Fig. S1c). This could be explained by the fact that Rsat II locus contains more tandem repeats than Rsat I. This pattern of continuous or partial rings around the NPBs was also observed in 2-cell stage embryos (asterisk in Fig. 1b and arrow in Fig. S1c), although the sequences were less decondensed than in pronuclei. At the 4-cell and early 8-cell stages, the Rsat I and II sequences started to cluster together (arrows in Fig. 1b) and were located in one part of the nuclei (Fig. S2) but were still associated with some NPBs (arrow Fig. S1c). From the 8- to 16-cell stages, the Rsat I and II sequences formed larger foci (arrowheads in Fig. 1b) which appeared to be distributed at random within the nuclei (Fig. 1b, lower panel). Still, some foci are found at the surface of some NPBs (arrow Fig. S1c).

**Fig. 2:**
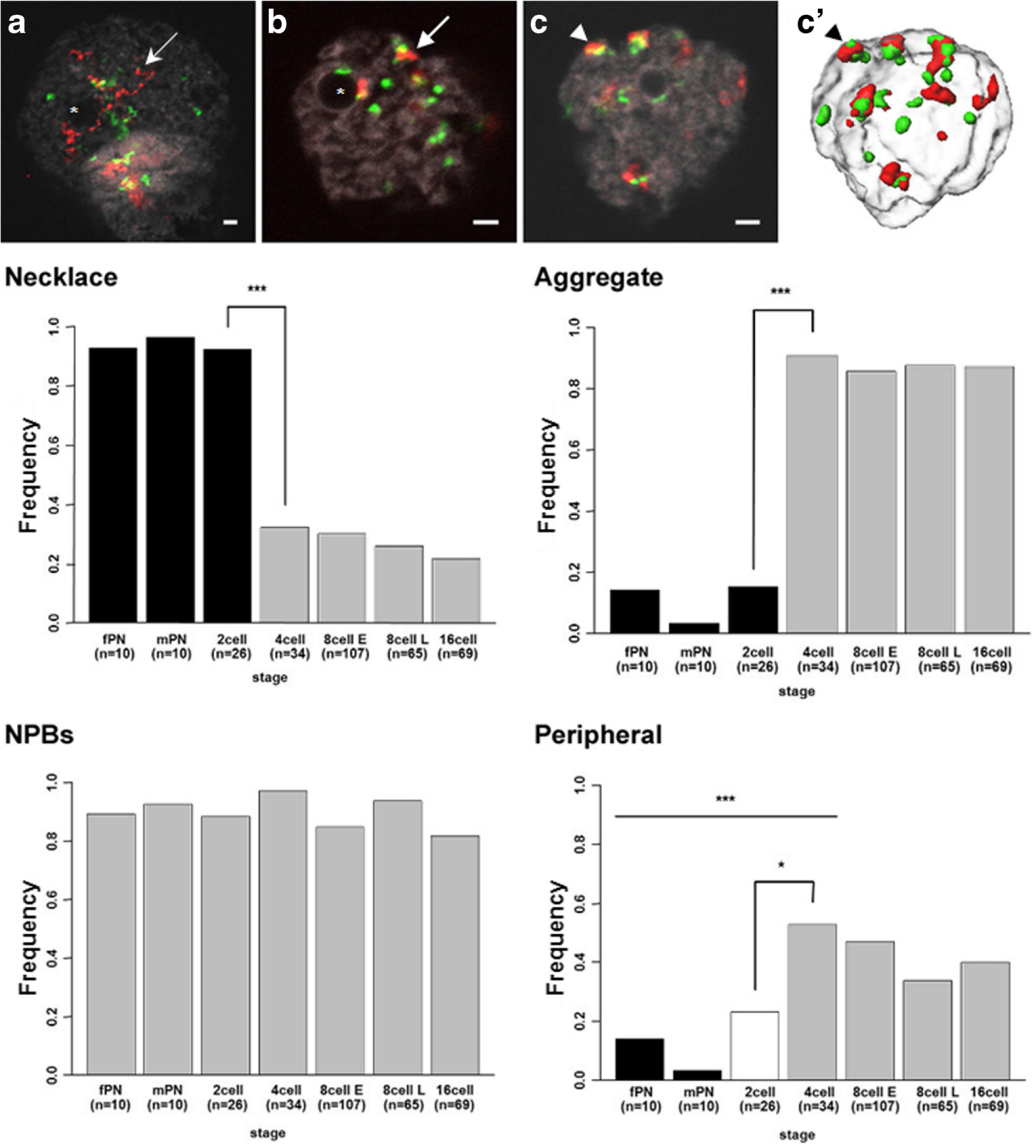
Spatial organization of Rsat I/II sequences during preimplantation development in the rabbit. **a–c**, **c′** Single confocal section of representative images (**a**–**c**) with Rsat I (green), Rsat II (red), and DNA (gray) labeling as well as the corresponding 3D reconstruction (**c′**) obtained using Amira software. These images illustrate the four parameters used to analyze the distribution of Rsat I/Rsat II FISH signals in the nucleus of rabbit embryos: (1) necklace (fine arrow in **a**), (2) aggregate (arrow in **b**), (3) NPBs (asterisks in **a**, **b**), and (4) peripheral (arrowheads in **c**, **c′**). Scale bar = 2 μm. *lower panel* Histograms corresponding to the frequencies of these four parameters at each stage of early development in the rabbit (1- to 16-cell stages). At the 1-cell stage, female and male pronuclei (mPN and fPN, respectively) are considered separately. The difference in frequencies between 2- and 4-cell stages for the necklace and aggregate parameters are highly significant (***p < 10^−5^). NPB parameter frequencies are homogenous throughout early development. Differences in the frequencies of the peripheral parameter are significant between fPN-mPN and the 4-cell stage (***p < 0.005) and less significant between the 2- and 4-cell stages (*p < 0.1)

**Fig. 3:**
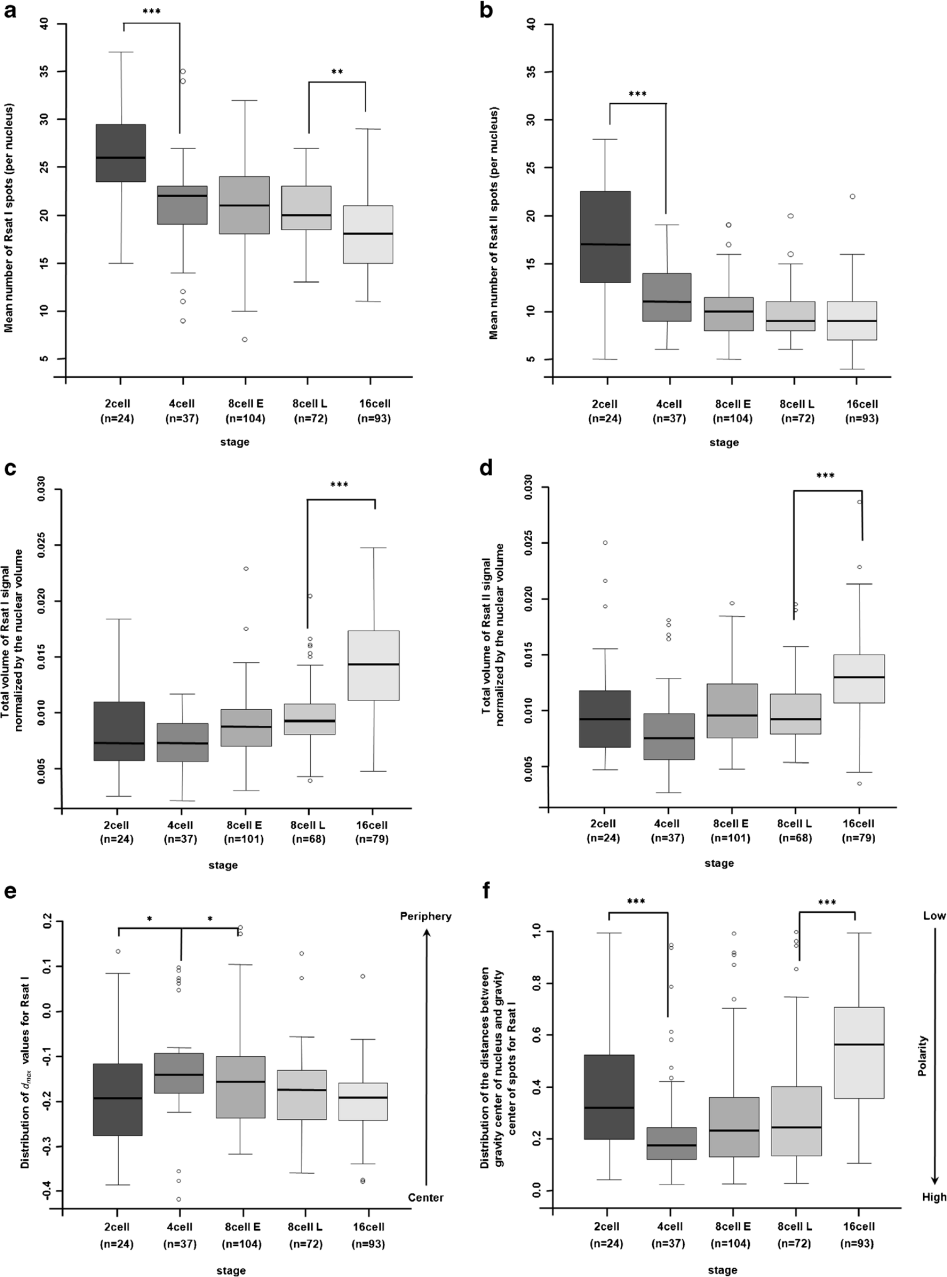
Quantitative automated analysis of Rsat I/Rsat II FISH signals in preimplantation rabbit embryos. Box plots indicate (per nucleus and per stage) the mean number of Rsat I (**a**) and Rsat II (**b**) spots and the fraction of the nuclear volume occupied by Rsat I/Rsat II FISH signals (**c**, **d**). Box plots (**e**) represent the maximum distance (*d*_max_) values between a theoretical uniform distribution and the observed distribution of EVF in nuclei. *d*_max_ values vary between − 1 and 1, with positive values indicating a bias towards a more peripheral signal, and negative values a more central one. Box plots (**f**) represent the distribution of distances between the centers of gravity of the nucleus and Rsat spots (values range from 0 to 1). This distribution allows us to evaluate the degree of polarity (high vs. low) of Rsat (I or II) signals in the nuclei at each developmental stage. The number of nuclei analyzed at each stage is indicated in brackets under the name of the stage. Significant differences in mean values between stages are indicated by asterisks (***p < 10^−5^, **p < 0.001, and *p < 0.05)

To further investigate the spatial and temporal distribution of these sequences, we defined four parameters to characterize the Rsat signal pattern (Fig. 2). These parameters were as follows: (1) Rsat I or II sequences forming pearl necklace-like structures (named “necklace”; fine arrow in Fig. 2a), (2) Rsat I or II sequences forming aggregates (named “aggregate”; arrow in Fig. 2b), (3) Rsat I or II sequences located at the periphery of the NPBs (highlighted by an asterisk in Fig. 2a, b or an arrow in Fig. S1c), and (4) Rsat I or II sequences located at the periphery of the nuclear envelope (named “peripheral”; arrowhead in Fig. 2c, c′). We quantified them in terms of their presence (1) or absence (0) at each stage. These parameters were not mutually exclusive.

As shown in Fig. 2, the pearl necklace organization of Rsat I and Rsat II sequences was characteristic of the 1- and 2-cell stages (Fig. 2, necklace). The percentage of nuclei with this structure exceeded 90% at these stages, then decreased significantly (*p* < 10^−5^) and rapidly at later stages to reach only ~20% at the 16-cell stage. Conversely, the organization of the signal in aggregates (Fig. 2, aggregate) was little present at the 1-cell stage and then increased significantly after the 2-cell stage (*p* < 10^−7^). Indeed, only ~15% of the nuclei had this configuration at the 2-cell stage, whereas this percentage reached more than 85% during subsequent stages. In fact, these two necklace and aggregate parameters displayed opposite behaviors and their frequencies switched significantly at the 4-cell stage, as shown in Fig. 2. The “NPB” parameter (Fig. 2, NPBs) did not vary during this period of embryonic development and a high percentage of the nuclei (85–97%) presented Rsat sequences associated with NPBs (arrows in Fig. S1c). Finally, the percentage of nuclei displaying an RsatI/RsatII distribution at the nuclear periphery (Fig. 2, peripheral) was very low (~14%) at the 1- and 2-cell stages when compared to later stages, the difference being significant (*p* = 0.0223). Moreover, at the 1-cell stage, fPN displayed a higher percentage signal (14.3%) at the periphery than mPN (3.6%), of minor significance (*p* = 0.0616). This percentage reached a maximum (~53%) at the 4-cell stage, and then decreased slightly during subsequent stages when fewer than 46% of the nuclei displayed this distribution (no significant difference).

### Spatial analyses of Rsat I/Rsat II sequence distribution during early development

The visual analysis data suggested that Rsat sequences were not randomly distributed but mainly located in the nuclear center, except at the 4-cell stage. As well as being more peripheral, Rsat signals also appeared to be restricted to one part of the nucleus, i.e., polarized. To test these hypotheses, we segmented 3D images of nuclei from post-zygotic embryonic stages (as described in the “Material and methods” section) to measure several nuclear shape parameters (volume, area, etc.).

From these data, we observed that nuclear volume decreased progressively (Fig. S3a) from the 2-cell (2794.47 μm^3^ ± 1136.91; *n* = 24) through the 16-cell stage (1152.17 μm^3^ ± 406.79; *n* = 93) as had previously been described during early embryonic development in the mouse (Aguirre-Lavin et al. 2012). Similarly, we segmented the Rsat I/Rsat II fluorescent signals that enabled a quantitative analysis of number of spots per nucleus. We then calculated the mean numbers of spots per nucleus and per stage (Table 1) and compared them to the number of spots expected as determined in metaphase (Fig. 1a), i.e., 24 spots for Rsat I and 12 spots for Rsat II. We also measured the total volume occupied by Rsat I and Rsat II FISH spots per nucleus (Fig. S3b, c) and calculated the mean volume of each spot for Rsat I and Rsat II FISH signals, respectively (Fig. S3d, e). These last measures (Fig. S3d, e) showed that Rsat II spots mean volume is always higher than Rsat I spots, which could be explained by a higher number of repeats (Ékes et al. 2004).

**Table 1:** Analysis of the number of Rsat I/II spots during preimplantation development in the rabbit

Stage	No. of nuclei studied	Rsat I		Rsat II	
		Mean ± SD	*p* value	Mean ± SD	*p* value
2-cell (27 hpc)	24	26.17 ± 5.08	0.04788	16.96 ± 6.10	0.000592
4-cell (34 hpc)	37	21.78 ± 6.57	0.04767	11.57 ± 3.34	*0.4358*
Early 8-cell (42 hpc)	104	20.96 ± 4.86	5.392e−09	10.04 ± 2.89	3.878e−10
Late 8-cell (49 hpc)	72	20.30 ± 3.44	1.49e−13	9.60 ± 2.41	2.241e−12
16-cell (58 hpc)	93	18.30 ± 3.56	2.2e−16	9.18 ± 2.78	7.552e−16

We performed Student’s *t* test to compare the theoretical number of Rsat I spots (μ0 = 24) or Rsat II spots (μ0 = 12) found on metaphase with the observed numbers of Rsat I or Rsat II spots counted automatically in the segmented nuclei of rabbit embryos at several stages. The *p* value in italic correspond to an alpha risk higher than 0.05 (the risk of rejecting the Null hypothesis when in fact it is true)

The mean number of Rsat I/Rsat II spots was statistically higher from that expected at the 2-cell stage but not at the 4-cell stage (Table 1). This could be due to the decondensed status of Rsat signals at early stages (Fig. 1), allowing the detection of signal doublets upon replication or leading to artefactual signal segmentation. Indeed, there was an overall decrease in the mean number of Rsat I/Rsat II spots from the 2- to 4-cell stage (Fig. 3a, b) that was highly significant. The total volume of both Rsat signals decreased from the 2- to 4-cell stage (Fig. S3b, c), suggesting a compaction of these sequences from the 4-cell stage.

At later stages, we observed fewer Rsat I/Rsat II spots than expected from the 8- to 16-cell stages (Table 1) and the reduction in the number of Rsat I spots was highly significant between the late 8- and 16-cell stages (*p* = 0.00108, Student’s *t* test). This reduction in the number of spots could be explained by aggregation of the Rsat signals. However, while the total volume of both Rsat signals increased slightly until the 16-cell stage (Fig. S3b, c), supporting this hypothesis, the mean spot volumes were constant for Rsat I as well as Rsat II whatever the stage (Fig. S3d, e). Since the volume of the nuclei also decreased during this period, we therefore examined Rsat volumes (I and II) normalized to the nuclear volume (Fig. 3c, d). We observed that this ratio remained constant from the 2- to 8-cell stage, independently of the Rsat sequence or the stage analyzed, and increased significantly at the 16-cell stage (*p* < 10^−11^). We concluded that Rsat I/Rsat II sequences aggregated from the 8-cell through the 16-cell stages, as illustrated by an increase in spot volume and a reduction in their number.

Next, we decided to determine the radial distribution of the signal using EVF methods. EVF is a normalized measurement of the radial position of the center of an object (see the “Material and methods” section). The EVF value ranged from 0 at the periphery to 1 at the center. EVF was calculated for each Rsat spot per nucleus (Fig. S4), and we observed that Rsat signals (independently of sequences and stages) were mostly detected in the center of the nucleus. In order to compare the results obtained in histogram form, we calculated the maximum algebraic distance (noted *d*_max_) between the observed EVF distributions of Rsat signals and a theoretical distribution function that followed the uniform law. We then used the Kolmogorov-Smirnov test to compare the distribution of *d*_max_ values between two stages and calculate the corresponding *p* values (Fig. 3e). Statistical analysis (linear model) of the *d*_max_ distribution confirmed that all Rsat signals were localized in the center of the nuclei (*p* values < 0.005; Fig. 3e) and showed that there were no significant differences between the distributions of Rsat I and II signals, although the latter were more central than those of Rsat I (*p* values > 0.1; data not shown). Statistical analysis (linear model) of Rsat signal distribution during development indicated a slight tendency to move towards the periphery at the 4-cell stage (*p* value = 0.016). The Rsat sequences then return to the center of the nuclei at the 8-cell stage (*p* value = 0.033).

We then determined whether the spots were polarized in the nuclei by comparing the distances between the center of gravity of the nucleus (called centroid) and that of the Rsat spots. We evaluated whether the distribution of this distance differed significantly from a theoretical distribution and calculated a *p* value (Fig. 3f). The greater the distance is (or the lower the *p* value is), the more polarized the signal is. As had previously been shown, there was no significant difference between Rsat I and II. All stages displayed statistically significant polarization (*p* values < 0.05) except the 16-cell stage. This polarization changed during development: it increased between the 2- and 4-cell stages (*p* value = 0.01) and then decreased between the 8- and 16-cell stages (*p* < 10^−12^). This quantitative analysis thus confirmed that the 4-cell (as observed in Fig. S2) and 8-cell stages displayed a more polarized state than all the other stages.

### Heterochromatin and NPB interactions during early development in the rabbit

**Fig. 4:**
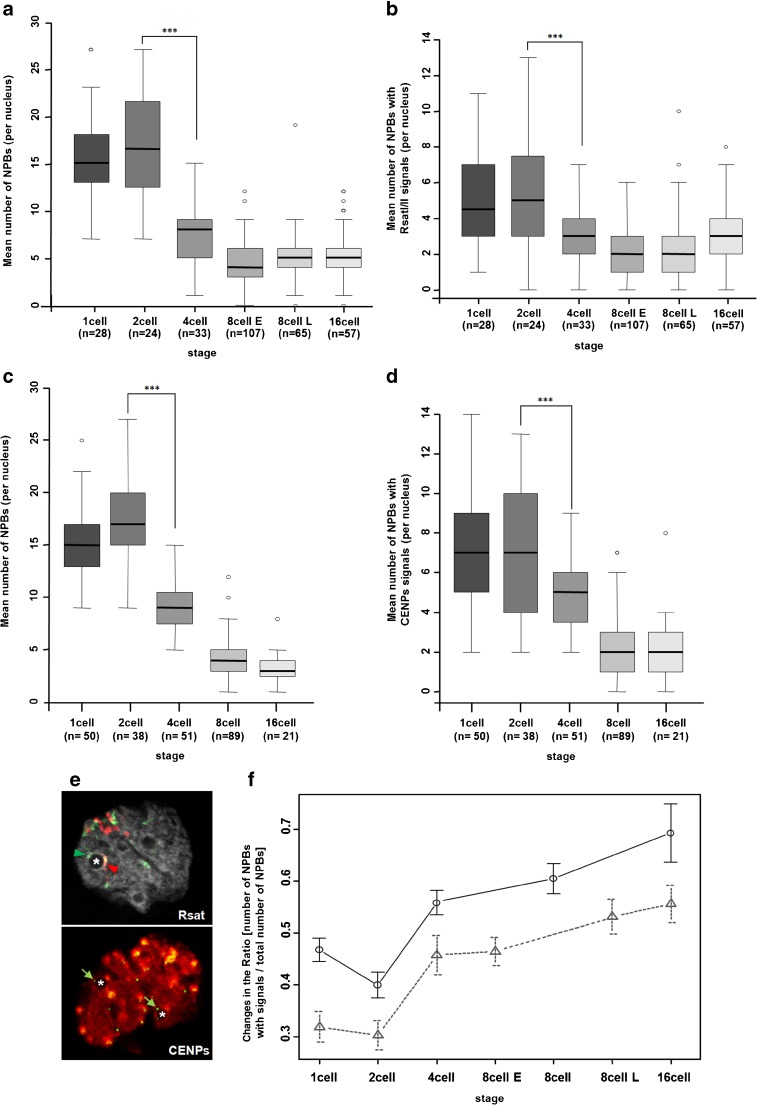
Quantitative analysis of the number of NBPs and associations with the pericentromeric/centromeric regions in preimplantation rabbit embryos. **a**, **b** Box plots indicating the total number of NPBs (**a**) and the number of NPBs associated with Rsat (I or II or both) FISH signals (**b**) counted in Rsat 3D images (representative NPBs are indicated with an asterisk in Fig. 1 and an arrow in Fig. S1c). **c**, **d** Box plots indicate the total number of NPBs (**c**) and the number of NPBs associated with CENP immunolabeled signals (**d**) counted in CENP/HP1β 3D images (Fig. S5). **e** Single confocal section of representative images of NPBs (indicated with an asterisk) associated with Rsat I (green arrowhead) and Rsat II (red arrowhead), in the upper panel, and associated with CENPs (green arrows), in the lower panel. *upper panel* Rsat I (green), Rsat II (red), and DNA (gray). *lower panel* CENPs (green) and HP1β (red). **f** This figure shows changes in the ratio between the number of NPBs associated with Rsat I/Rsat II signals (dashed line with triangle markers) or CENP signals (solid line with circle markers) and the total number of NPBs during preimplantation development. The number of nuclei analyzed at each stage is indicated in brackets under the name of the stage. Significant differences in mean values between stages are indicated by asterisks (***p < 10^−5^)

As previously noted, at least one NPB was located close to the Rsat sequences at all stages. Therefore, in order to clarify a possible link between NPB and Rsat sequence organization, we counted the total number of NPBs and the number of NPBs associated with Rsat sequences. We observed a pronounced decrease in the total number of NPBs from the 2-cell through the 16-cell stage (Fig. 4a). We found that the number of NPBs with (at least one) Rsat FISH signal at its periphery (asterisk in Fig. 1b, arrows in Fig. S1c, and arrowheads in Fig. 4e) decreased from the 2-cell (5.17 ± 2.96) through the 4-cell stage (3.18 ± 1.68) (Fig. 4b). However, the number of NPBs with signals divided by the total number of NPBs increased during development (gray line in Fig. 4f), suggesting that NPBs without Rsat signals were disappearing.

Rsat I and II probes did not label all the chromosomes, so we also analyzed the distribution of HP1β and CENPs at the same stages. In rabbit embryos, HP1β foci appeared and started to associate with CENP dots at the 4-cell stage. The clustering of HP1β associated with CENP dots reached a maximum at the 8-cell stage (Fig. S5; Yang et al. 2013). We evaluated the number of NPBs associated with CENP dots at their periphery (asterisk in Fig. S5 and green arrows in Fig. 4e) in 1-cell through 16-cell stage embryos. As observed on the 3D-FISH images, the total number of NPBs decreased significantly from the 2-cell through the 16-cell stages (*p* = 4.511e−08) (Fig. 4c). The number of NPBs associated with CENP dots fell from a mean number of 7.14 (± 2.80) at the 1-/2-cell stages to a mean number of 2.28 (± 1.62) at the 16-cell stage (Fig. 4d). As described for Rsat sequences, the normalized mean number of NPBs associated with CENP dots increased throughout development (Fig. 4f, black line).

The mean number of NPBs per stage analyzed using the two different signals (Rsat FISH and CENP) differed significantly at the 4-, 8-, and 16-cell stages between the two types of image, but followed the same trend. We did not observed any significant difference between the slopes of the two linear curves (*p* = 0.985; gray and black lines in Fig. 4f) that represent the number of NPBs associated with Rsat FISH signals and the number of NPBs associated with CENP signals, both normalized to the total number of NPBs.

Overall, NPB interactions with heterochromatin regions appeared to change in shape during very early development in the rabbit. The Rsat sequences were organized in pearl necklace structures wrapping the NPBs during the first two developmental stages (Fig. 1 and Fig. S1c), and then formed smaller clusters during subsequent stages (Figs. 1 and 2). Although still interacting with NPBs up to the 16-cell stage, the surface of interaction between these sequences and the NPBs seems to decrease.

**Fig. 5:**
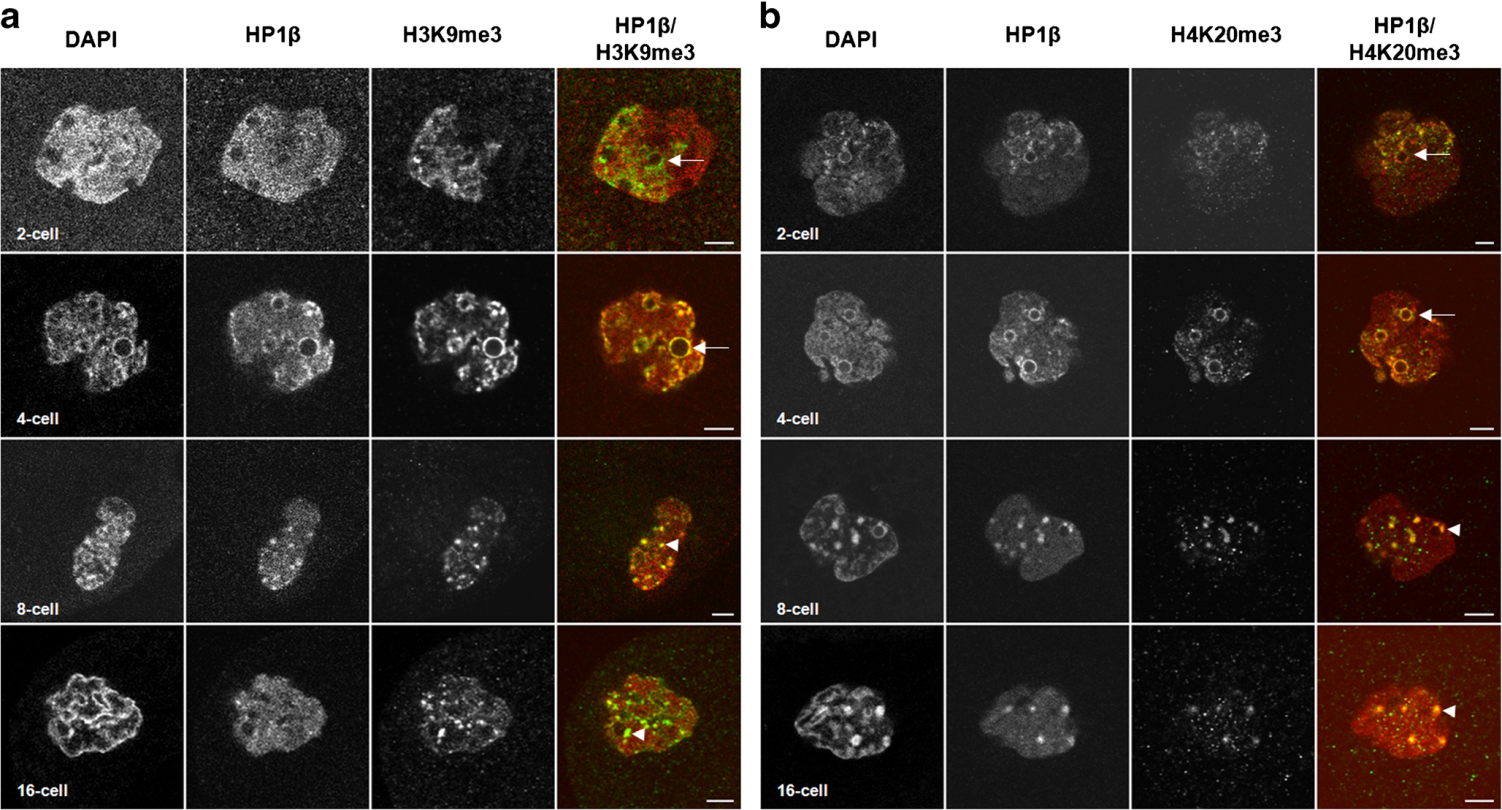
Spatial localization of H3K9me3 and H4K20me3 during rabbit preimplantation development. Single confocal section of representative images of nuclei from embryos fixed at the 2-cell (24 hpc), 4-cell (34 hpc), 8-cell (49 hpc), and 16-cell (58 hpc) stages. Scale bar = 5 μm. Arrow indicates an accumulation of the stained protein around NPB. Arrowhead indicates an accumulation of the stained protein forming clusters. *left panel* Immunostaining of H3K9me3 (green) and HP1β (red). *right panel* Immunostaining of H4K20me3 (green) and HP1β (red)

To further investigate the behavior of these pericentromeric regions, we performed immunostaining with two classical heterochromatin histone marks (H3K9me3 and H4K20me3) concomitantly with HP1β on whole-mount embryos from the 1-cell through the 16-cell stages (Fig. S6 and Fig. 5).

As expected, H3K9me3 staining specifically labeled the female chromatin (fPN) at the 1-cell stage (Reis e Silva et al. 2011; Fig. S6, upper panel) and was restricted to one side of the nucleus at the 2-cell stage (Fig. 5a). H3K9me3 surrounded NPBs at the 2- and 4-cell stages (Fig. 5a, arrows) and co-localized with HP1β clusters at the 8- and 16-cell stages (Fig. 5a, arrowheads). Similarly, we only found H4K20me3 signal in the fPN at the 1-cell stage (Fig. S6, lower panel). Then, the signal was located in one part of the nucleus at the 2-cell and was even brighter around the NPBs at the 4-cell stage (Fig. 5b, arrow). From the 8-cell through the 16-cell stage, all the nuclei displayed H4K20me3 patches which co-localized partially with HP1 clusters (Fig. 5b, arrowhead). Taken together, these data suggest that epigenetic modifications affect these regions during the very early stages of development.

## Discussion

During this study, we examined the spatial and temporal distribution of pericentromeric heterochromatin regions in rabbit embryos labeled with fluorescence probes specific to Rsat I and Rsat II sequences over the course of early embryonic development (from the 1- to 16-cell stage), using the 3D image computational analysis of various nuclear parameters (nuclear volume, Rsat volume, NPB number, etc.).

### Spatial organization of pericentromeric heterochromatin regions and EGA

Our results showed that Rsat sequences (i) exhibited a “bead on a string” structure at the 1- and 2-cell stages; (ii) started to compact at the 4-cell stage, i.e., just before EGA; and (iii) aggregated at the 8- and 16-cell stages, i.e., during and after EGA. We also found that independently of the stage, Rsat sequences displayed a central position in the nuclei (although at the 4-cell stage, their location was closer to the periphery). More intriguing was that these sequences were not randomly distributed in the nucleus. Statistical analysis underlined the fact that Rsat sequences were polarized and significantly located in one part of the nuclei at the 4-cell and early 8-cell stages. These results agreed with the data previously obtained by our laboratory using a similar approach on early-stage mouse embryos (Aguirre-Lavin et al. 2012). These changes to the spatial organization of Rsat sequences at the 4-cell stage in the rabbit may be linked to the context of major EGA at the 8-cell stage that might require heterochromatin reshaping before that time. Indeed, in the mouse, decompaction of the pericentromeric regions occurs when minor transcription is activated (1-cell stage) and ends with EGA (at the 2-/4-cell stages). Interestingly, other studies (Probst et al. 2010; Casanova et al. 2013) demonstrated the importance of pericentromeric sequence transcription at the end of the zygotic stage and during the 2-cell stage which enables developmental progression beyond the 2-cell stage. They also showed that major satellite transcripts are required for the reorganization of pericentromeric heterochromatin regions in chromocenters (Probst et al. 2010). Given these findings in the mouse, it might be interesting to test whether Rsat sequences are transcribed during early embryonic stages (before and after EGA) in the rabbit, and whether inhibition of their transcription might be detrimental to further development.

### Nuclear volume and NPBs in rabbit embryos

In a recent study, Popken and colleagues showed that nuclear volume in rabbit embryos decreases from the 2-cell through the blastocyst stages (Popken et al. 2016). We found similar results during our study using different analytical methods. Furthermore, previous studies in mouse (Aguirre-Lavin et al. 2012) and bovine (Popken et al. 2015) embryos had demonstrated a similar reduction in average nuclear volume during early development. These findings allowed us to postulate that this phenomenon is conserved among mammalian species. In addition, Popken et al. (2016) demonstrated a major remodeling of the nuclear envelope during early development in the rabbit. More precisely, they revealed a peak of nuclear membrane invaginations that were positive for lamin B and Nup153 (a nucleoporin that participates in formation of the nuclear pore complex) at the 4-cell stage. They speculated that these invaginations ensure proximity to the cytoplasm for NPBs and might reflect a considerable need for proteins at this particular stage, concomitantly with the first step of nucleologenesis (Baran et al. 1997). Similar to our previous study in the mouse (Aguirre-Lavin et al. 2012), we found that the overall number of NPBs decreased at the 4-cell stage and even more drastically at the 8-cell stage, when rRNA transcription starts (Baran et al. 1997). Our observations also revealed that pericentromeric regions always interacted with NPBs but that these interactions changed in shape over the course of development. Indeed, pericentromeric heterochromatin region sequences reorganized themselves from a pearl necklace distribution surrounding NPBs into a cluster distribution juxtaposed to the NPBs. Thus, even if the pericentromeric heterochromatin regions were still in interaction with NPBs, the amount of interactions appeared to decrease at the 4-cell stage of development. Taken together, these findings tempt us to speculate that important changes occur at the 4-cell stage. These changes may link nucleogenesis and heterochromatin organization, as was already suggested by a recent study in mouse embryos (Fulka and Langerova 2014).

### Epigenetic features of heterochromatin in the rabbit

In this study, we were able to confirm our previous data demonstrating the distribution of HP1β and CENPs during rabbit development in fertilized and cloned embryos (Yang et al. 2013). While HP1β presented a diffuse pattern in the nucleoplasm from the 1- to 2-cell stages, RsatI/II distribution was much more dispersed, as described above. We can therefore assume that there was no correlation between the pattern of HP1β immunostaining and the location of Rsat sequences at these stages. More HP1β foci, or even patches, were observed at the 4-cell stage, but no chromocenter-like structure could be seen in the rabbit embryonic cell nucleus before the 8-cell stage (at the time of EGA). We presume that Rsat sequences may mainly correspond to pericentromeric regions rather than centromeric regions, and that CENPs allow monitoring of the behavior of centromeric regions.

We questioned whether pericentromeric heterochromatin regions in the rabbit followed the same rules as pericentromeric heterochromatin regions in the mouse (Santenard et al. 2010; Beaujean 2014) or bovine (Pichugin et al. 2010). We therefore examined the distribution of H3K9me3 and H4K20me3 (two classic markers of constitutive heterochromatin) from the 1-cell through the 16-cell stage.

As demonstrated in the mouse (Santos et al. 2005) and bovine (Pichugin et al. 2010), we also found that H3K9me3 labeled the maternal genome in rabbit zygotes (Reis e Silva et al. 2011). In the mouse, this mark is particularly enriched at the maternal pericentric heterochromatin (Probst et al. 2007; Puschendorf et al. 2008; Tardat et al. 2015). However, the signal was more dispersed in rabbit embryos, although rings around the NPBs could sometimes be detected (arrow in Fig. 5a). On the other hand, the asymmetry of H3K9me3 between the paternal and maternal genomes was clearly detectable up to the 4-cell stage, which is similar to findings in the mouse (Beaujean 2002; Mason et al. 2012) and bovine (Pichugin et al. 2010). Furthermore, as was previously shown in the mouse (Martin et al. 2006a) and bovine (Pichugin et al. 2010), we found that the H3K9me3 signal gradually co-localized with HP1β patches or clusters at the time of EGA.

In mouse and human somatic cells, the tri-methylation on lysine 20 of histone 4 (H4K20me3) is localized primarily at centromeres, pericentromeres, and telomeres (Schotta et al. 2008). The present study is the first that describes H4K20me3 during early development in the rabbit. H4K20me3 staining was very similar to H3K9me3 staining. As in mouse, this histone mark was found only on the female pronucleus at the 1-cell stage. However, this mark was quite dispersed in the rabbit female pronucleus, while in the mouse, H4K20me3 signal was found only around NPBs (Probst and Almouzni 2008; Eid et al. 2016). After the 1-cell stage, comparison with the mouse is more complicated. We found contradictory data in the literature: some authors (Wongtawan et al. 2011; Eid et al. 2016) could not detect H4K20me3 after the 1-cell stage, whereas others (Puschendorf et al. 2008; Ancelin et al. 2016) have shown, in supplementary data, staining of H4K20me3 in 2-cell mouse embryos. These differences could be due to the antibody used to detect H4K20m3. When we performed immunostaining experiments in rabbit embryos with the same antibodies (Fig. 5b and Fig. S6, lower panel; data not shown), we observed H4K20me3 signal from the 1-cell through the 16-cell stages. Interestingly, H4K20me3 condensed and formed clusters partially co-localized with HP1β at the 8-cell stage, the EGA stage in rabbit, thus correlating with the H4K20me3 staining observed on chromocenters in 2-cell mouse embryos (Fig. S7; Puschendorf et al. 2008; Ancelin et al. 2016). This pattern could be explained by the fact that H4K20me3 is deposited by SUV4–20H which is recruited by HP1α, and binds to H3K9me3 (Kourmouli et al. 2004; Schotta et al. 2004).

## Conclusion

In conclusion, we have shown that Rsat I and Rsat II sequences change in terms of their localization and compaction between the 2- and 4-cell stages. The radial distribution and polarization of these pericentromeric sequences differ significantly at the 4-cell stage when compared to other stages of early embryonic development. We also observed that the interaction between heterochromatin and NPBs was important at the start of development (2- and 4-cell stages) and was then modified after EGA.

Taken together, these results highlight the importance of the 4-cell stage as a transition point. This now requires further investigation in order to decipher the architectural changes required for proper embryonic genome activation in the rabbit, and to compare them with other species that undergo major genomic activation after several cell cycles (such as humans and bovine).

## Electronic supplementary material

Figure S1 **Spatial distribution of Rsat I/Rsat II FISH signals in the nucleus of a fibroblast (A), in the nucleus of a 4-cell stage embryo (B) and their association with NPBs (C).** 3D-FISH experiments were performed with specific probes for Rsat I (green) and Rsat II (red). DNA was counterstained with Yopro-1 (gray). Scale bar = 5 μm. Upper right panel: (**A**) Single confocal section of a representative image of a fibroblast nucleus in the three dimensions: xy, yz and xz. The “xz” image shows that Rsat I/Rsat II FISH signals are located at the periphery. Lower right panel: (**B**) Single confocal section of a nucleus of a 4-cell stage embryo in the three dimensions (xy, yz and xz). We observed that the thickness of the fibroblast nucleus (~5 μm) was smaller than that of the nucleus of the 4-cell embryo (~13 μm). Left panel (**C**) Single confocal section of representative images of a nucleus from embryos fixed at 1-cell stage (19 h post-coïtum (hpc) with female and male pronuclei (fPN and mPN), and at 2-cell (24hpc), 4-cell (34hpc), early and late 8-cell (42 and 49hpc respectively) and 16-cell (58hpc) stages. Arrows indicate NPBs associated with either Rsat I or Rsat II FISH signals or both. (GIF 78 kb)

High resolution image (TIFF 2733 kb)

Figure S2 **Example of the spatial distribution of Rsat I/Rsat II FISH signals in all nuclei of a 4-cell rabbit embryo.** 3D-FISH experiments were performed on a 4-cell embryo fixed at 34 h post-coitum (hpc) with specific probes for Rsat I (green)/Rsat II (red). DNA was counterstained with Yopro-1 (gray). Full Z-series projections (maximal intensity) are shown. Images were adjusted for brightness/contrast settings in each individual channel using ImageJ. The dotted lines (white) show a hypothetical boundary in the sequence distribution. Scale bar = 5 μm. (GIF 44 kb)

High resolution image (TIFF 1950 kb)

Figure S3 **Quantitative automated analysis of nuclear and Rsat I/Rsat II signal volume in preimplantation rabbit embryos.** Box plots presented here correspond to the variation of the volume of the nucleus (assess with DNA staining) (A), the total volume (per nucleus) of Rsat I (B) and Rsat II (C) FISH signals and the mean volume of Rsat I (D) and Rsat II (E) spots from the 2-cell to the 16-cell stage embryos in rabbit. The number of nuclei analyzed at each stage is indicated in brackets under the stage. At the 8-cell stage, early (E) and late (L) embryos (before and after embryonic genome activation) were analyzed separately. Differences in mean nuclear volume values (A) between each stage were highly significant (*p* < 10^−6^) except between the early and late 8-cell stages. Nuclear volume decreased at each cell division. However, the total volume of the Rsat (I or II) FISH signal first decreased significantly between the 2-cell and 4-cell stages and then remained constant. (GIF 46 kb)

High resolution image (TIFF 8185 kb)

Figure S4 **Distribution of EVF values.** These histograms represent the distribution of EVF values at each stage (2-cell, 4-cell, early 8-cell, late 8-cell and 16-cell) for Rsat I (A) and Rsat II (B) signals. (GIF 30 kb)

High resolution image (TIFF 4474 kb)

Figure S5 **Spatial organization of CENPs and HP1β proteins during rabbit preimplantation development.** Immunostaining of CENPs proteins (green) and HP1β (red). Scale bar = 5 μm. Left panel: Single confocal section of representative images of nuclei from embryos fixed at the 2-cell (24 hpc), 4-cell (34 hpc), 8-cell (49 hpc) and 16-cell (58 hpc) stages. Right panel: Z-maximal projections of representative images of a nucleus from the same 3D images. HP1β is diffused in the nucleoplasm at the 2-cell and 4-cell stages. At the 8-cell stage, HP1β proteins accumulate in a cluster associated with at least one CENP dot. (GIF 90 kb)

High resolution image (TIFF 5.35 kb)

Figure S6 **Spatial localization of H3K9me3 and H4K20me3 at the 1-cell stage in the rabbit embryo.** Female and male pronuclei are indicated as fPN and mPN, respectively. A single confocal section and z-maximal projection of representative images are presented here. Scale bar = 5 μm. Upper panel: Immunostaining of H3K9me3 (green) and HP1β (red) in a zygote. Only the maternal pronucleus is labeled by H3K9me3. Lower panel: Immunostaining of H4K20me3 (green) and HP1β (red) in a zygote. Only the maternal pronucleus is labeled by H4K20me3. The punctuated circle on z-maximal projection delimits paternal pronucleus. (GIF 107 kb)

High resolution image (TIFF 3072 kb)

Figure S7 **Spatial localization of H4K20me3 during mouse preimplantation development.** Single confocal section of representative images of nuclei from embryos fixed at the 2-cell (44hphCG), 4-cell (56hphCG), 8-cell (62hphCG) and 16-cell (76hphCG) stages. Scale bar = 5 μm. Arrow indicates an accumulation of the stained protein around NPB. Arrowhead indicates an accumulation of the stained protein forming clusters. DAPI (gray), H4K20me3 (green) and HP1β (red). (GIF 2.57 kb)

High resolution image (TIFF 7365 kb)

## Abbreviations

3D: Three-dimensional
BSA: Bovine serum albumin
CENPs: Centromeric proteins A/B
DAPI: 4′,6-Diamidino-2-phenylindole
EGA: Embryonic genome activation
EVF: Eroded volume fraction
FISH: Fluorescent in situ hybridization
FITC: Fluorescein isothiocyanate
H3K9me3: Histone-3 tri-methylated in lysine 9
H4K20me3: Histone-4 tri-methylated in lysine 20
HP1: Heterochromatin protein 1
NPB: Nucleolar precursor body
PBS: Phosphate-buffered saline
PFA: Paraformaldehyde
mPN: Male pronucleus
fPN: Female pronucleus
RT: Room temperature
Rsat: Rabbit satellite sequences
SSC: Saline sodium citrate buffer
