# Supplementary figures and images for: Three-dimensional analysis of nuclear heterochromatin distribution during early development in the rabbit
Source: Chromosoma. 2018 Apr 18;127(3):387–403. doi: 10.1007/s00412-018-0671-z (PMC6096579; doi:10.1007/s00412-018-0671-z)

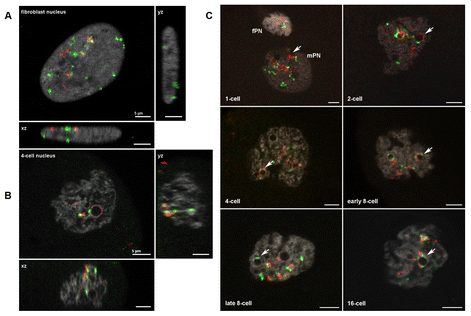

Supplement: Supplementary file 1 — Spatial distribution of Rsat I/Rsat II FISH signals in the nucleus of a fibroblast (A), in the nucleus of a 4-cell stage embryo (B) and their association with NPBs (C). 3D-FISH experiments were performed with specific probes for Rsat I (green) and Rsat II (red). DNA was counterstained with Yopro-1 (gray). Scale bar = 5 μm. Upper right panel: (A) Single confocal section of a representative image of a fibroblast nucleus in the three dimensions: xy, yz and xz. The “xz” image shows that Rsat I/Rsat II FISH signals are located at the periphery. Lower right panel: (B) Single confocal section of a nucleus of a 4-cell stage embryo in the three dimensions (xy, yz and xz). We observed that the thickness of the fibroblast nucleus (~5 μm) was smaller than that of the nucleus of the 4-cell embryo (~13 μm). Left panel (C) Single confocal section of representative images of a nucleus from embryos fixed at 1-cell stage (19 h post-coïtum (hpc) with female and male pronuclei (fPN and mPN), and at 2-cell (24hpc), 4-cell (34hpc), early and late 8-cell (42 and 49hpc respectively) and 16-cell (58hpc) stages. Arrows indicate NPBs associated with either Rsat I or Rsat II FISH signals or both. (GIF 78 kb) [file 412_2018_671_Fig6_ESM.gif]

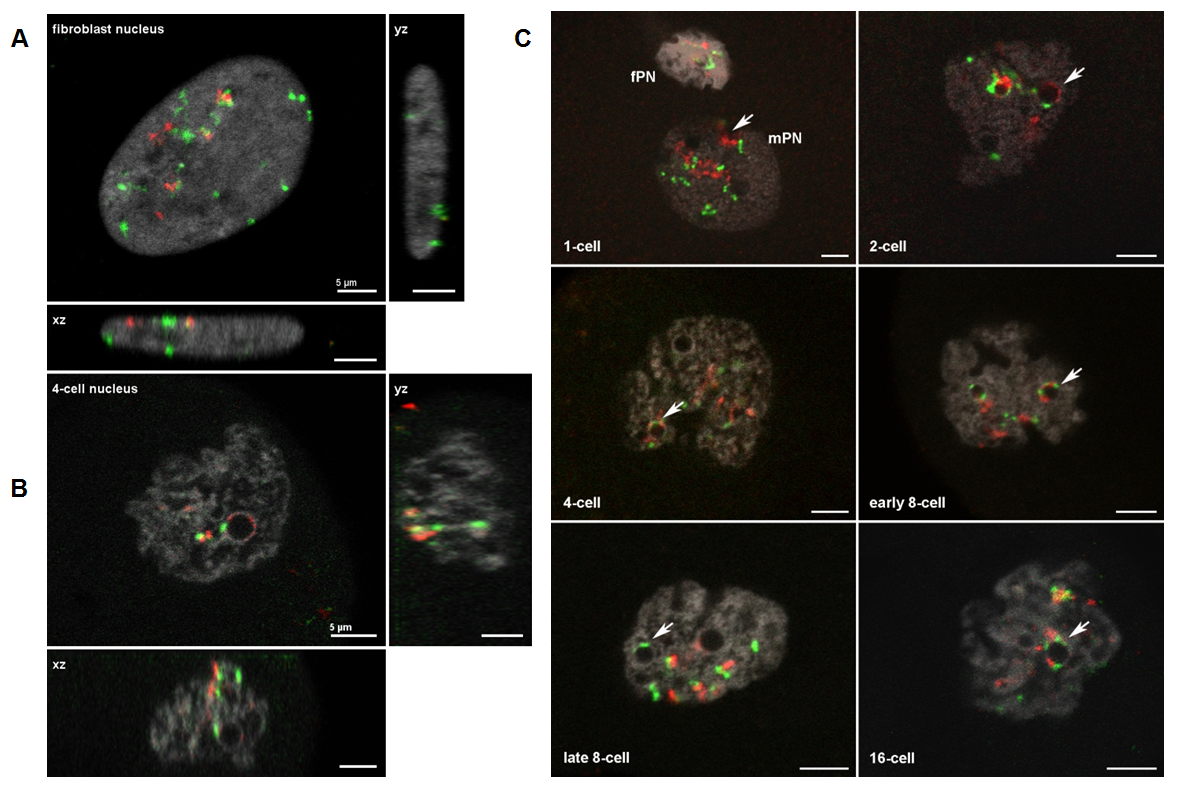

Supplement: Supplementary file 2 — High resolution image (TIFF 2733 kb) [file 412_2018_671_MOESM1_ESM.tif]

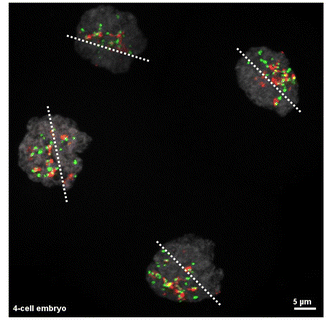

Supplement: Supplementary file 3 — Example of the spatial distribution of Rsat I/Rsat II FISH signals in all nuclei of a 4-cell rabbit embryo. 3D-FISH experiments were performed on a 4-cell embryo fixed at 34 h post-coitum (hpc) with specific probes for Rsat I (green)/Rsat II (red). DNA was counterstained with Yopro-1 (gray). Full Z-series projections (maximal intensity) are shown. Images were adjusted for brightness/contrast settings in each individual channel using ImageJ. The dotted lines (white) show a hypothetical boundary in the sequence distribution. Scale bar = 5 μm. (GIF 44 kb) [file 412_2018_671_Fig7_ESM.gif]

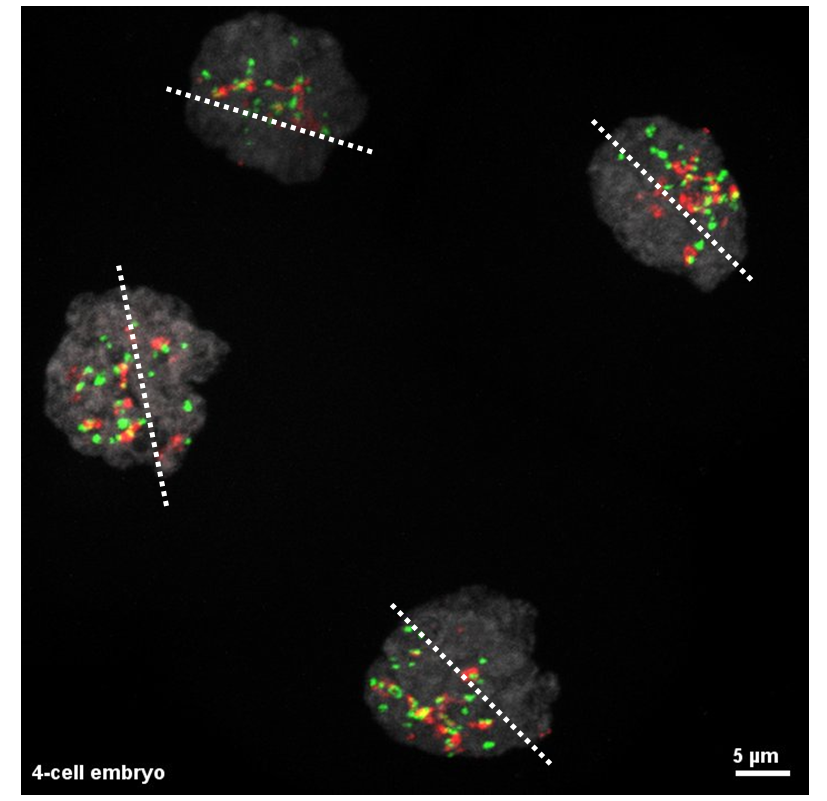

Supplement: Supplementary file 4 — High resolution image (TIFF 1950 kb) [file 412_2018_671_MOESM2_ESM.tif]

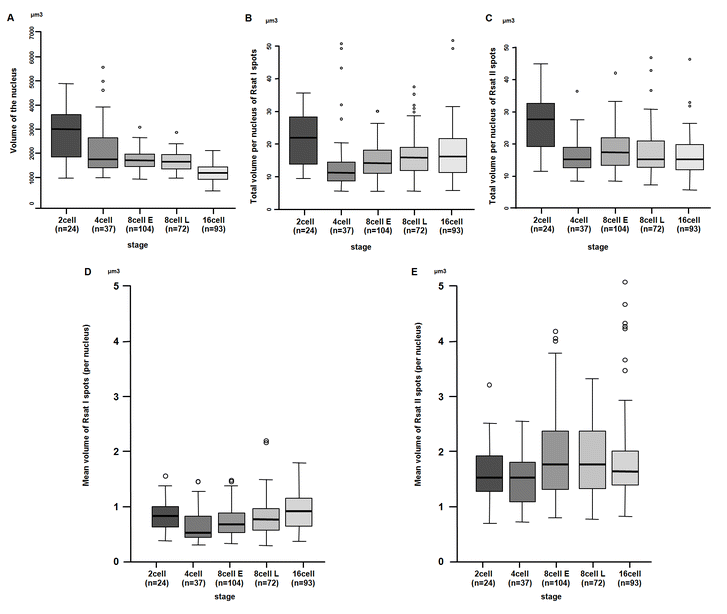

Supplement: Supplementary file 5 — Quantitative automated analysis of nuclear and Rsat I/Rsat II signal volume in preimplantation rabbit embryos. Box plots presented here correspond to the variation of the volume of the nucleus (assess with DNA staining) (A), the total volume (per nucleus) of Rsat I (B) and Rsat II (C) FISH signals and the mean volume of Rsat I (D) and Rsat II (E) spots from the 2-cell to the 16-cell stage embryos in rabbit. The number of nuclei analyzed at each stage is indicated in brackets under the stage. At the 8-cell stage, early (E) and late (L) embryos (before and after embryonic genome activation) were analyzed separately. Differences in mean nuclear volume values (A) between each stage were highly significant (p < 10−6) except between the early and late 8-cell stages. Nuclear volume decreased at each cell division. However, the total volume of the Rsat (I or II) FISH signal first decreased significantly between the 2-cell and 4-cell stages and then remained constant. (GIF 46 kb) [file 412_2018_671_Fig8_ESM.gif]

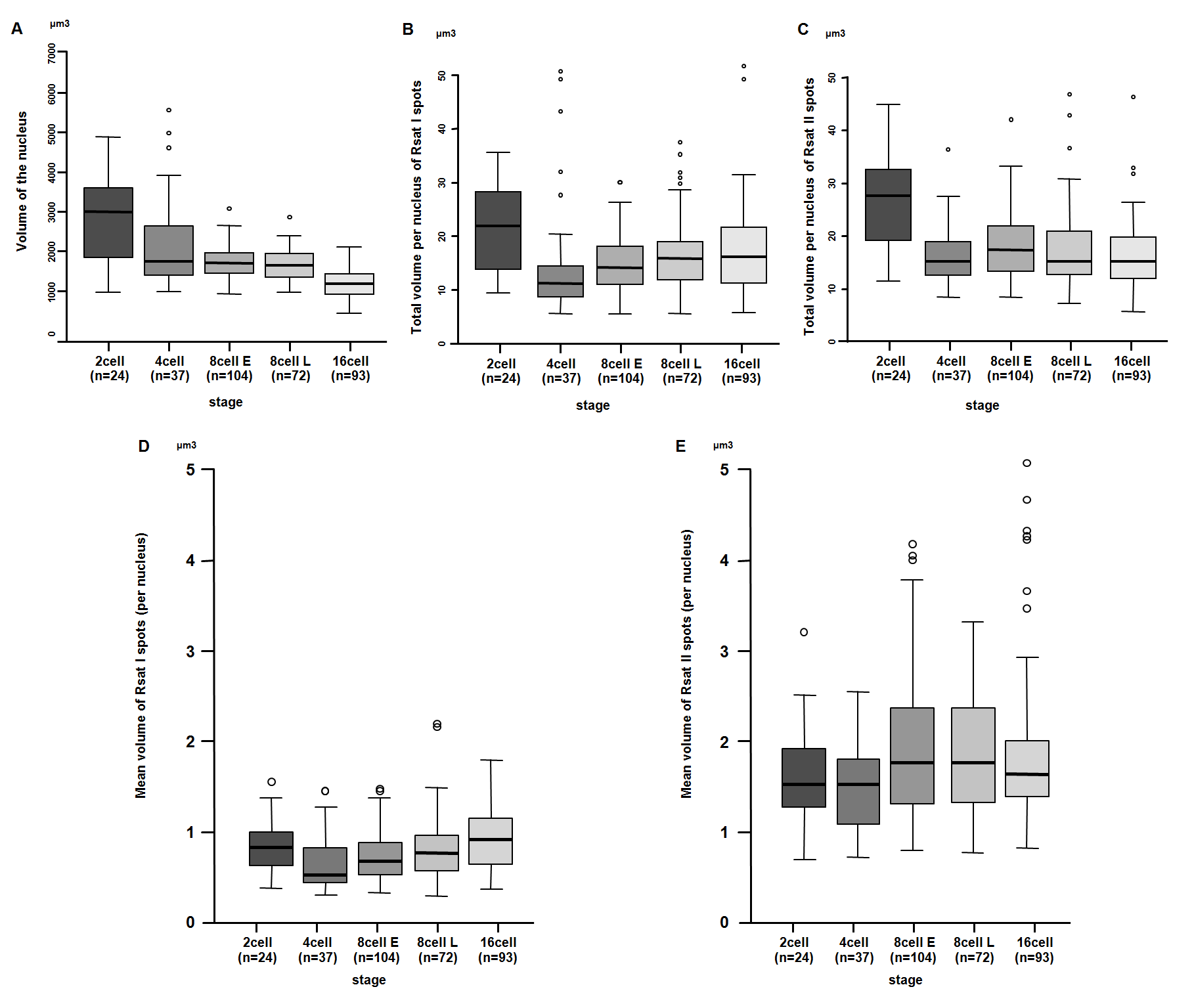

Supplement: Supplementary file 6 — High resolution image (TIFF 8185 kb) [file 412_2018_671_MOESM3_ESM.tif]

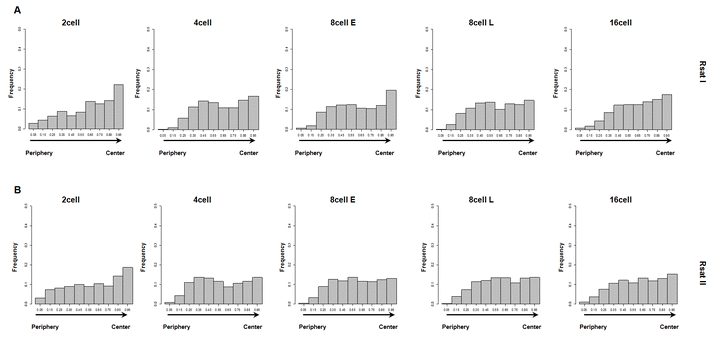

Supplement: Supplementary file 7 — Distribution of EVF values. These histograms represent the distribution of EVF values at each stage (2-cell, 4-cell, early 8-cell, late 8-cell and 16-cell) for Rsat I (A) and Rsat II (B) signals. (GIF 30 kb) [file 412_2018_671_Fig9_ESM.gif]

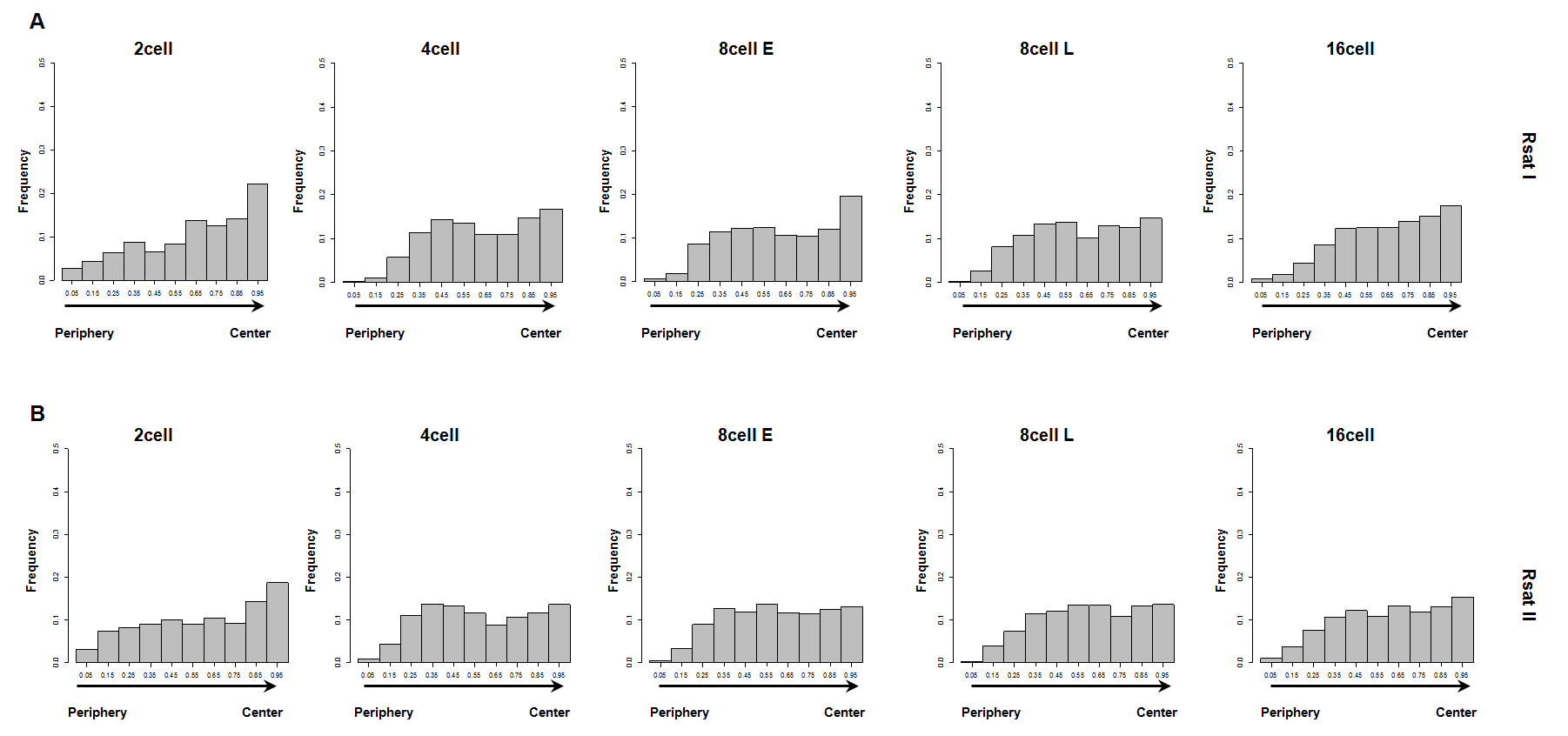

Supplement: Supplementary file 8 — High resolution image (TIFF 4474 kb) [file 412_2018_671_MOESM4_ESM.tif]

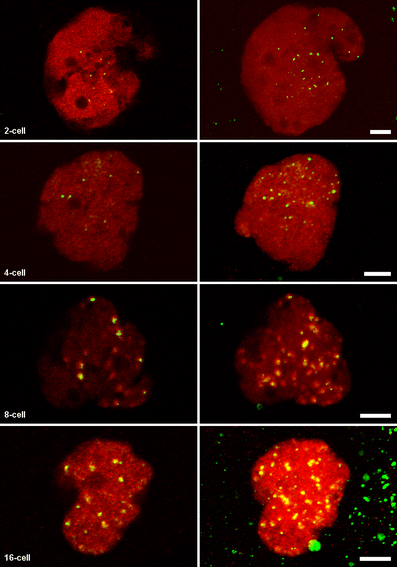

Supplement: Supplementary file 9 — Spatial organization of CENPs and HP1β proteins during rabbit preimplantation development. Immunostaining of CENPs proteins (green) and HP1β (red). Scale bar = 5 μm. Left panel: Single confocal section of representative images of nuclei from embryos fixed at the 2-cell (24 hpc), 4-cell (34 hpc), 8-cell (49 hpc) and 16-cell (58 hpc) stages. Right panel: Z-maximal projections of representative images of a nucleus from the same 3D images. HP1β is diffused in the nucleoplasm at the 2-cell and 4-cell stages. At the 8-cell stage, HP1β proteins accumulate in a cluster associated with at least one CENP dot. (GIF 90 kb) [file 412_2018_671_Fig10_ESM.gif]

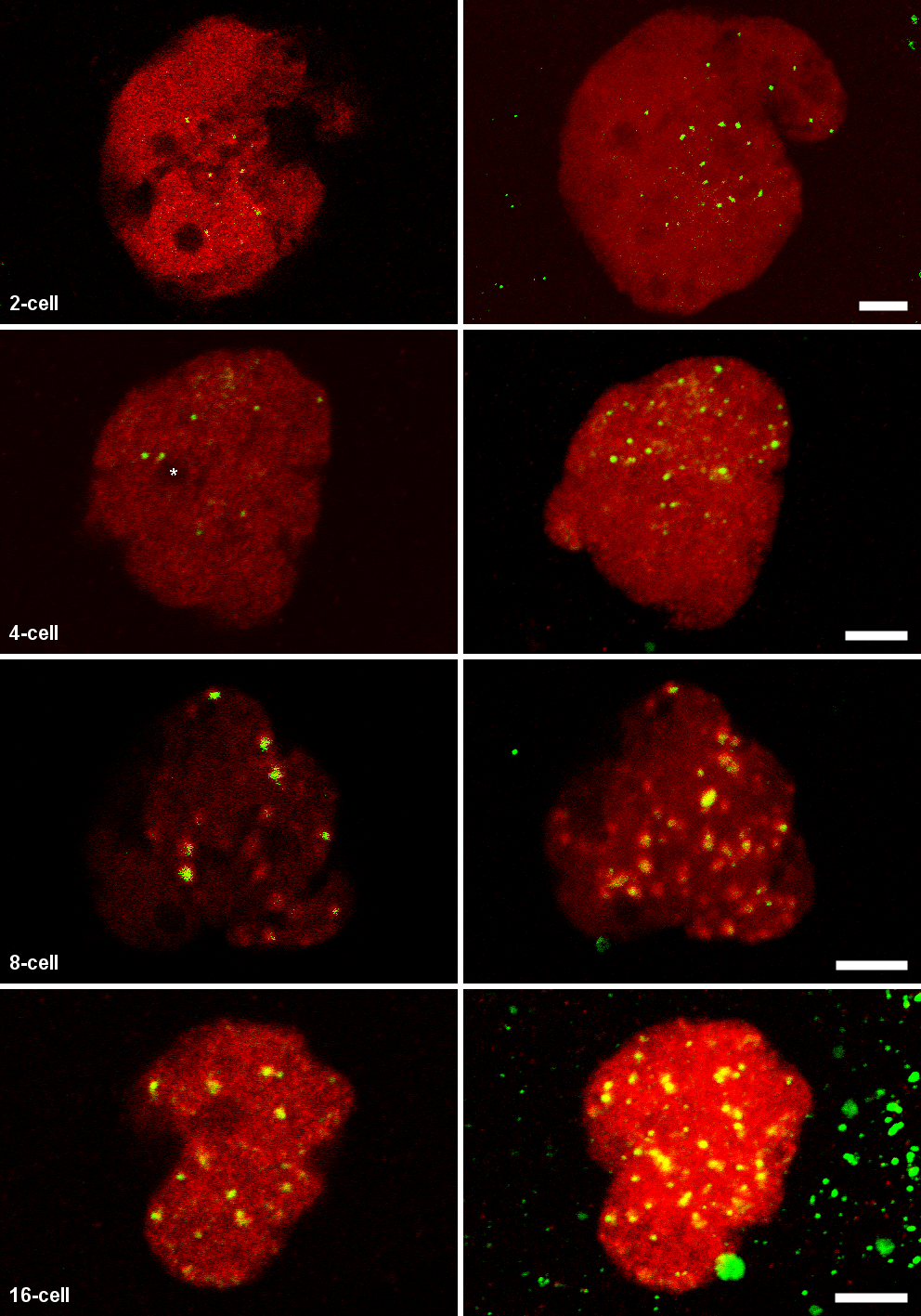

Supplement: Supplementary file 10 — High resolution image (TIFF 5.35 kb) [file 412_2018_671_MOESM5_ESM.tif]

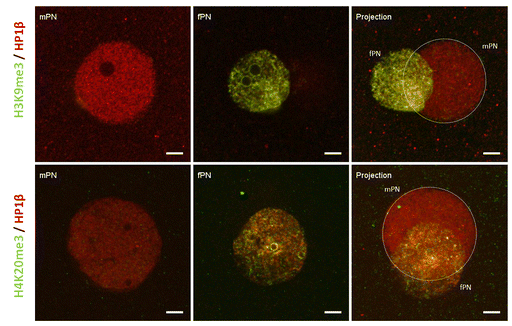

Supplement: Supplementary file 11 — Spatial localization of H3K9me3 and H4K20me3 at the 1-cell stage in the rabbit embryo. Female and male pronuclei are indicated as fPN and mPN, respectively. A single confocal section and z-maximal projection of representative images are presented here. Scale bar = 5 μm. Upper panel: Immunostaining of H3K9me3 (green) and HP1β (red) in a zygote. Only the maternal pronucleus is labeled by H3K9me3. Lower panel: Immunostaining of H4K20me3 (green) and HP1β (red) in a zygote. Only the maternal pronucleus is labeled by H4K20me3. The punctuated circle on z-maximal projection delimits paternal pronucleus. (GIF 107 kb) [file 412_2018_671_Fig11_ESM.gif]

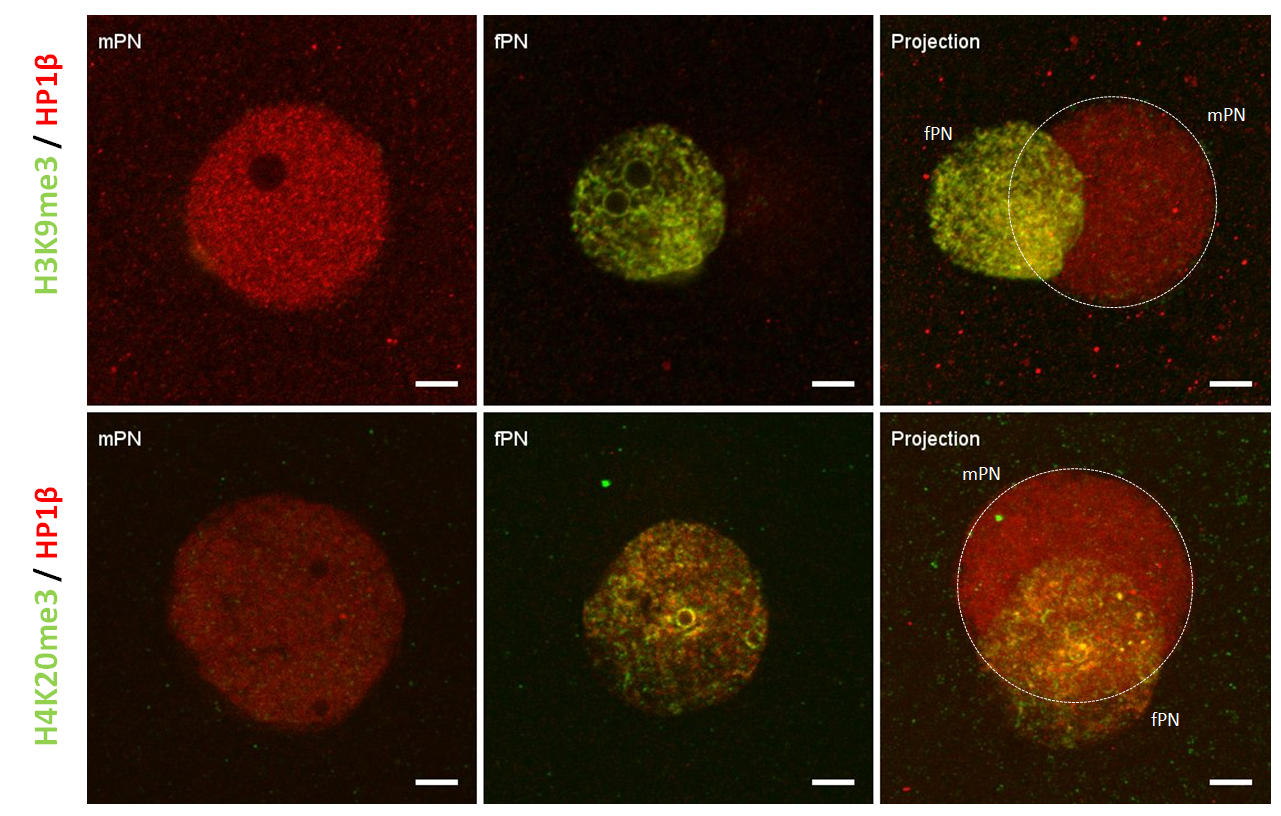

Supplement: Supplementary file 12 — High resolution image (TIFF 3072 kb) [file 412_2018_671_MOESM6_ESM.tif]

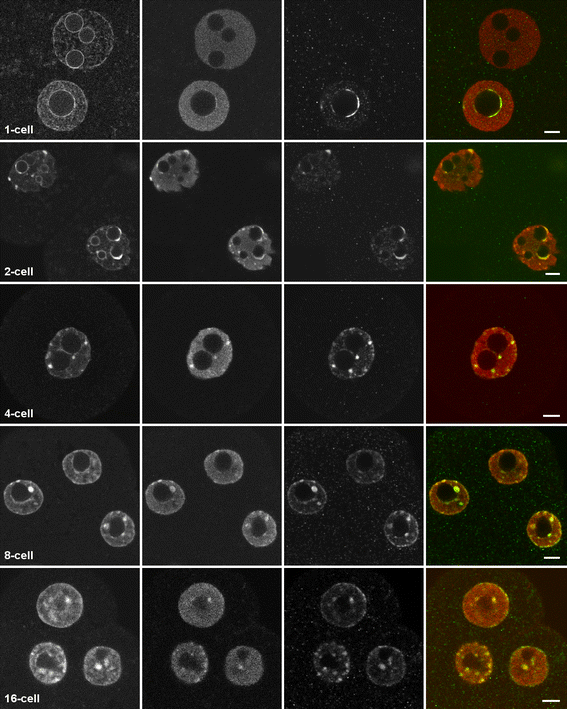

Supplement: Supplementary file 13 — Spatial localization of H4K20me3 during mouse preimplantation development. Single confocal section of representative images of nuclei from embryos fixed at the 2-cell (44hphCG), 4-cell (56hphCG), 8-cell (62hphCG) and 16-cell (76hphCG) stages. Scale bar = 5 μm. Arrow indicates an accumulation of the stained protein around NPB. Arrowhead indicates an accumulation of the stained protein forming clusters. DAPI (gray), H4K20me3 (green) and HP1β (red). (GIF 2.57 kb) [file 412_2018_671_Fig12_ESM.gif]

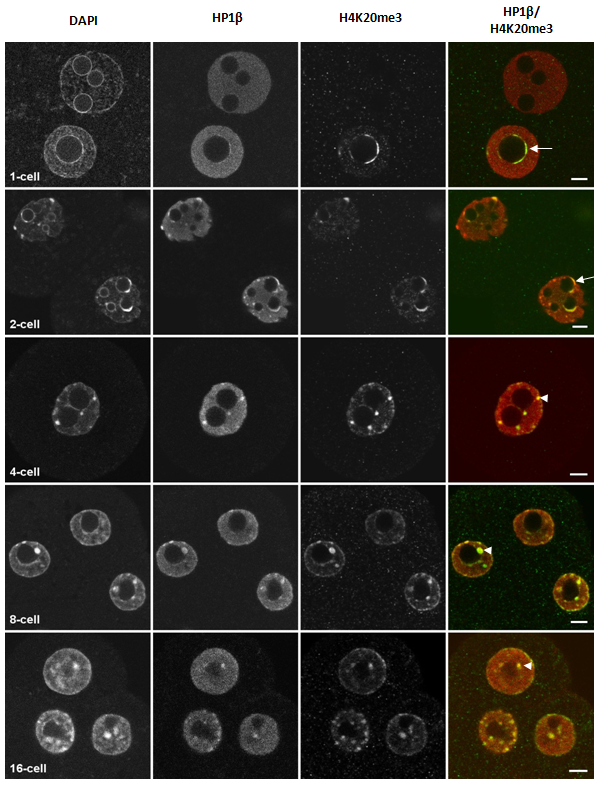

Supplement: Supplementary file 14 — High resolution image (TIFF 7365 kb) [file 412_2018_671_MOESM7_ESM.tif]
